# A Review of the Regulatory Role of Plant Growth–Promoting Rhizobacteria in Alfalfa Under Stress Conditions

**DOI:** 10.3390/plants14213248

**Published:** 2025-10-23

**Authors:** Yu-Yan Zhang, Jin-Lei Liu, Xuan Wang, Xin Cao, Kang-Hui Liu, Yu-Ting Luo, Jia-Yin Chen, Jiang Zhang, Yong-Hong Fan

**Affiliations:** School of Life Science & Technology, Xinjiang University, Urumchi 830049, China; zhangyy@xju.edu.cn (Y.-Y.Z.); ljl1223@stu.xju.edu.cn (J.-L.L.);

**Keywords:** alfalfa, plant growth–promoting bacteria, nitrogen fixation capacity, saline and alkaline stress

## Abstract

Alfalfa (*Medicago sativa* L.) is a crucial plant for saline and alkaline soil development, which is crucial for managing the salinization of global land resources. It can withstand saline and alkaline stress and can fix nitrogen. By secreting phytohormones, fixing nitrogen, and boosting antioxidant capacity, nitrogen–fixing bacteria, rhizobacteria, and other inter–root biotrophic bacteria encourage alfalfa development and reduce salinity stress. Alfalfa’s symbiotic association also encourages other plants to tolerate salinity and greatly boosts the amount of nitrogen in the soil. The mechanism by which inter–root growth–promoting bacteria mitigate saline and alkaline stress in alfalfa remains a prominent research focus. This paper reviews the current state of research on inter–root probiotic bacteria associated with alfalfa, utilizing literature mining to summarize the resource information of inter–root nitrogen–fixing bacteria found in saline–alkaline soils. We elucidate their nitrogen-fixing mechanisms and adaptive characteristics, explore their roles and potential applications in the improvement of saline–alkaline lands, and provide a theoretical foundation for the development of novel nitrogen–fixing bacterial fertilizers and restoration technologies for saline–alkaline environments.

## 1. Introduction

Global land salinity has surpassed one billion hectares, significantly constraining agricultural production and undermining ecosystem stability [1,2,3]. This issue represents an increasingly serious challenge to the sustainable use of global land resources [4,5,6]. Soil salinity not only diminishes soil fertility and crop yields but also adversely impacts soil microbial diversity, which subsequently influences the stability and functionality of soil ecosystems [7,8,9].

Alfalfa is a significant leguminous forage crop known for its exceptional salinity tolerance and nitrogen–fixing capabilities. This plant not only enhances the fertility of saline soils through biological nitrogen fixation but also effectively mitigates the adverse effects of salinity stress on plant growth. Consequently, alfalfa demonstrates considerable potential for application in the improvement of saline lands and ecological restoration [10,11,12,13,14,15].

During the growth of alfalfa, plant growth–promoting bacteria (PGPR) interact with the plant’s root system, significantly enhancing both the growth and development of the plant, as well as its resistance to various stresses [16,17]. In this process, PGPR synergize with microorganisms, such as nitrogen–fixing bacteria, to enhance the nitrogen fixation efficiency of alfalfa. This interaction is crucial for improving the soil environment [18]. However, significant shortcomings remain in the current research concerning the regulatory mechanisms of alfalfa inter–root–promoting bacteria under saline and alkaline stress. Additionally, the relationship between microbial community structure and function, as well as the prospects for practical application, has not been thoroughly and systematically discussed.

This study aims to review the current status of the regulation of alfalfa rhizobacteria under saline and alkaline stress by synthesizing existing research findings. It explores the interaction mechanisms between alfalfa and rhizobacteria, revealing the pivotal role of rhizobacteria in enhancing alfalfa’s tolerance to saline and alkaline conditions as well as its nitrogen fixation capacity. Furthermore, the study analyzes the potential applications of these interactions in improving saline and alkaline lands, providing a theoretical foundation and practical guidance for the development of new nitrogen–fixing bacterial fertilizers and technologies for the restoration of saline and alkaline soils. This paper aims to provide both theoretical foundations and practical guidance for the development of novel nitrogen–fixing bacterial fertilizers and the restoration of saline–alkaline lands. By integrating scientific research with applied methodologies, this study seeks to enhance agricultural productivity and sustainability in challenging environments.

## 2. Current Status of Alfalfa Research

Currently, research on alfalfa primarily concentrates on the mechanisms of salt tolerance, the biological enhancement of saline–alkaline soils, growth mechanisms, and the selection and breeding of salt-tolerant varieties [19]. The study of salt tolerance and the biological enhancement of saline and alkaline land are the primary subjects of this work.

Soil salinization poses a hazard to about 954 million hectares of saline land worldwide [4,20]. Agro–ecosystem security and soil health are seriously threatened by soil salinization, which is a major limiting factor in the use of land resources. Over one billion hectares of land in China have become salinized, making up around 10% of the country’s total land area. This trend is growing annually, and the Songnen Plain in Northeast China, one of the three main regions in the world where pure alkaline saline is distributed, is currently in danger of getting worse due to the salinization of 3.73 million hectares of land [4,21,22]. The detrimental effects of saline soil on plant–soil systems are mostly caused by the combination of an alkaline climate and high salt. The primary mechanisms of action are as follows: (1) ionic toxicity, whereby an excess of Na^+^ and Cl^−^ damages the homeostatic balance of vital elements like K^+^ and Ca^2+^ through competitive uptake, causing disruptions in plant ion metabolism; (2) osmotic stress, where a water potential imbalance in the cell plasma membrane inhibits stomatal movement and photosynthetic electron transfer; and (3) oxidative stress, where an abnormal buildup of reactive oxygen species (ROS) causes membrane lipid peroxidation, which denaturates proteins and damages the structure of chloroplasts [23]. In summary, salt stress predominantly arises from the accumulation of neutral salts (e.g., NaCl and Na_2_SO_4_) in the soil. Elevated salt concentrations decrease soil water potential, thereby inducing osmotic stress that impedes water uptake by plants [24,25]. In contrast, alkali stress is mainly driven by high levels of alkaline salts, such as Na_2_CO_3_ and NaHCO_3_, which significantly increase soil pH [26]. The resulting alkaline conditions adversely affect the availability and absorption of essential mineral nutrients–particularly phosphorus, iron, and manganese–leading to ion imbalance and nutrient deficiency in plants [27].

In the end, these cascading responses cause plants to have dysregulated energy metabolism, decreased photosynthetic efficiency, and poor nitrogen uptake. Salinity hinders the breakdown of organic matter and the cycling of nutrients by drastically reducing the variety of microbial communities and decreasing the activity of important soil enzymes like urease and phosphatase [28,29]. In addition to lowering crop yields, this systemic damage causes soil fertility to continuously decline. To solve the salinization–induced crisis in soil health, a comprehensive management system founded on ecological control is desperately needed. Because of its ecological sustainability and ecologically friendly qualities, biological management has emerged as the go–to method for improving soil, according to research on saline–alkaline land restoration technology systems [30]. Among its main benefits are the following (1) The mechanism of microbial–plant synergy: Through biological nitrogen fixation, nitrogen–fixing bacteria increase the efficiency of plants’ nitrogen uptake while also stimulating the production of endogenous salt-resistant genes [17]; (2) The mechanism of nutrient regulation: Technology for distributing nitrogen fertilizer can maximize the C/N ratio of soil, encourage the mineralization of organic matter, and alter nitrogen forms [31,32]; and (3) The microbial efficiency mechanism: By secreting active ingredients such as 1–aminocyclopropane–1–carboxylic acid (ACC) deaminase, PGPR can systematically control plant osmotic adjustment and ionic homeostatic balance [31,32,33,34]. Building a three–in-one bioremediation system of “salt-tolerant plants–PGPR–nutrient regulation” has a higher chance of achieving a synergistic improvement in soil health and crop productivity than using a single technology pathway, such as physical or chemical.

**Figure 1:**
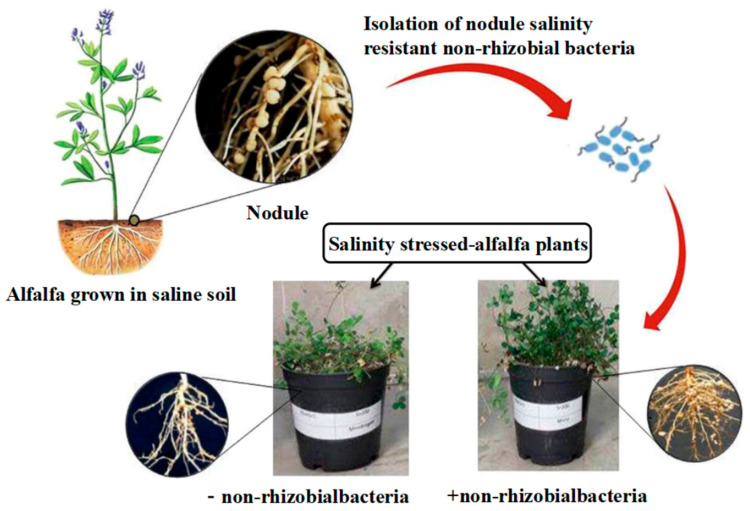
Alfalfa growing in saline and alkaline land [38].

Known as the “king of grasses” [10,11,31] (Figure 1), alfalfa is a typical representative of perennial leguminous herbaceous plants with ecological value and agronomic characteristics of double significance. It has been widely used to improve saline soils due to its good nitrogen–fixing capacity and ecological restoration valueImprovement of alkaline and saline land [11,31,33,34]. With three main benefits, it has emerged as a key crop in the production of fodder worldwide: (1) broad-spectrum adaptability: superior resilience in environments that are saline, semi-arid, and dry; (2) effective symbiotic nitrogen fixation: rhizobacterial interactions for atmospheric nitrogen biotransformation; and (3) superior forage qualities: it is a vital part of the ruminant feed system, with a crude protein level of 21.9% at [33,35]. The North American growing belt, the Eurasian temperate zone, and the Mediterranean climate zone are the three main production areas that have developed globally as a result of this biological property. The United States is the largest producer, making up more than 44% of the total area harvested from the U.S. crop in 2022 [36,37]. Alfalfa serves as both pasture production and ecological restoration in China’s agro–ecosystem. Its planting range includes the middle and lower sections of the Yangtze River Basin, the salty and alkaline regions of Northeast China, the North China Plain, and the desert region of Northwest China. In particular, alfalfa is extending its ecological service functions beyond traditional pasture to saline and alkaline land improvement, soil and water conservation, and has emerged as a significant bio–carrier for the sustainable development of agricultural and pastoral intertwined zones, all thanks to the promotion of the “changing grain to fodder” policy that was introduced in 2016.

**Figure 2:**
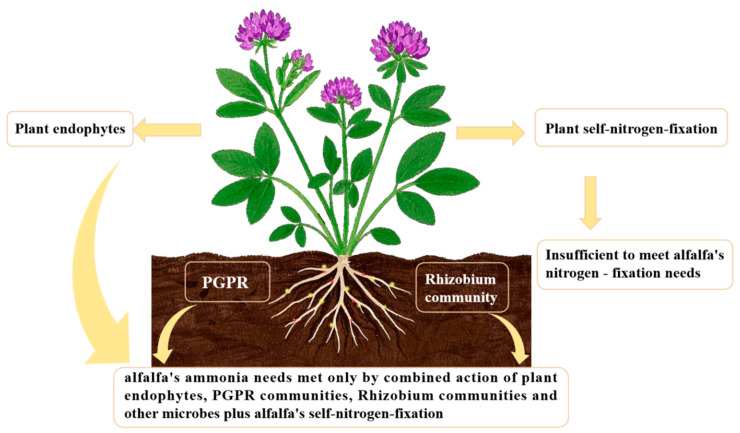
Mechanism of nitrogen fixation by alfalfa.

Research on soil development has focused on the ecological role of alfalfa’s inter–root microbial system because it is a pioneering plant that can withstand salinity and is used in agriculture. According to studies, the plant and inter–root multifunctional probiotic bacteria form a unique bioremediation system. On the one hand, the symbiotic flora directly supports the host’s growth by fixing nitrogen, solubilizing phosphorus, and secreting plant growth regulators (such as ACC deaminase, iron carriers, and extracellular polysaccharides, among others) [39,40,41]. However, the flora also demonstrates the capacity to degrade biopesticides, creating a mechanism for the degradation of pollutants that is benign to the environment. According to Liu et al.’s successful isolation of salt-tolerant and biotolerant strains from saline alfalfa’s inter–root, these microorganisms [42] not only increase the host’s biomass accumulation in stressful environments but also lessen salt damage through mechanisms like lowering the concentration of Na^+^ between roots. Additionally, using these microorganisms as a biofertilizer greatly lowers the environmental loading of chemical nitrogen fertilizers. By using it as a biofertilizer, the environmental impact of chemical nitrogen fertilizers was greatly decreased. It is noteworthy that alfalfa’s nitrogen metabolism displayed multifaceted symbiotic traits. Several nitrogen–fixing microorganisms, such as the *Actinobacteria* phylum and the thick–walled bacteria phylum, were also found at the inter–root level by Noori F’s team using macro–genomics analysis [43], even though the traditional rhizobial symbiosis system can supply a portion of the nitrogen source. Through synergistic effects, these flora increased the host nitrogen–fixing efficiency by 37–42%. This multiple symbiotic mechanism greatly increased the soil’s organic matter content and optimized the granular structure. It was confirmed (Figure 2) that the plant’s nitrogen–fixing enzyme activity could only satisfy 28–35% of its nitrogen demand, with the remaining amount relying on the metabolic compensation of the inter–root flora. The mechanism of the interaction between alfalfa and inter–root nitrogen–fixing bacteria was comprehensively evaluated in this work, which was based on research data from the previous 20 years and the advances in multi–omics technology during the last 5 years. By examining the system’s dual role in saline–alkaline land improvement–improving soil microbiology and increasing plant resistance–it offers a theoretical foundation for the creation of an ecological agriculture model centered on the cooperative activity of microorganisms and plants. In particular, it creates a new avenue for the sustainable use of salinized arable land in arid and semi-arid regions.

## 3. Plant–Microbe Interactions

### 3.1. Importance of Plant–Microbe Interactions

**Figure 3:**
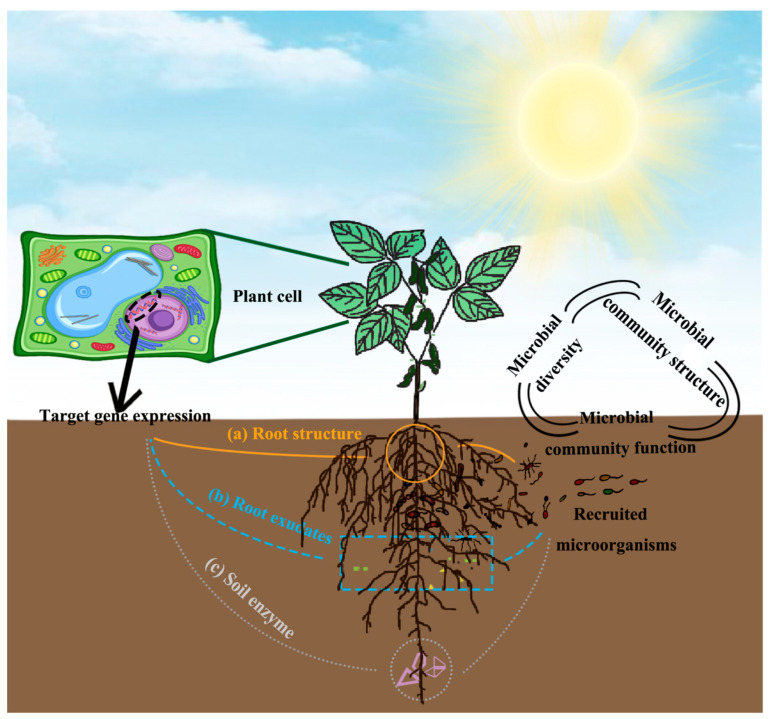
Diversity and function of inter–root microorganisms [47]. (**a**) Relevant plant genes, which typically control the synthesis of materials necessary for root morphology at the genetic and transcriptional levels, govern the development of root morphology (e.g., root length and number of lateral roots). The assembly of inter–root microbial communities is somewhat impacted by changes in root architecture, which implies variations in root feeding capacity; (**b**) plant functional genes affect inter–root microbial diversity and structure by regulating root secretions (e.g., phenolics, flavonoids, and hormones). (**c**) Soil enzyme activities, which are intimately linked to microorganisms, are regulated by the expression of host-specific genes.

**Figure 4:**
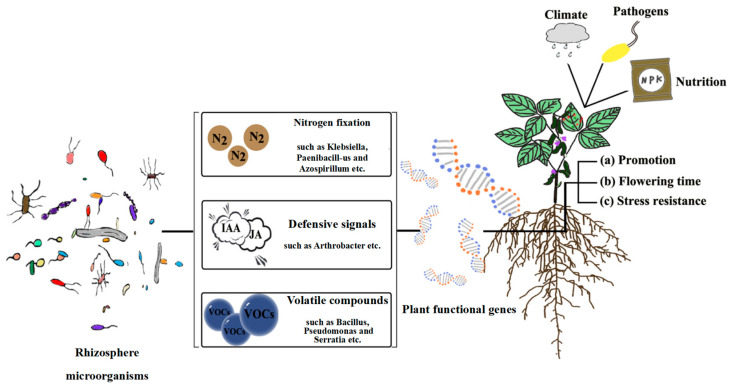
Mechanisms associated with inter–root microbes influencing plant growth and development, flowering time, and stress tolerance [47] (**a**) Microbes improved the biological fixation of N_2_. Microorganisms alter the content of various material components of the nitrogen cycle by stimulating the nitrogen cycle genes in the host, which in turn compensates for the material elements that the host is deprived of. In this way, microorganisms promote the nutrient uptake of the host and enable it to thrive. In addition to perceiving root secretions from plants, microbes themselves are capable of secondary metabolism to secrete specific substances, such as volatile compounds and hormones. Among them, volatile compounds have been shown to be direct drivers of plant growth hormone synthesis genes and photosynthesis genes. With the addition of plant growth hormones and photosynthesis, the growth of plants is affected. (**b**) Rhizosphere microorganisms directly or indirectly regulate the flowering time of plants. On the one hand, microbes secrete indole acetic acid (IAA) to directly regulate flowering genes to influence flowering time; on the other hand, microbes indirectly influence flowering time by constraining plant nutrient requirements and secreting volatile compounds. (**c**) Under biotic and abiotic stresses, microorganisms influence the expression of defence genes (e.g., climate, pathogens, and nutrient deprivation), which in turn activate defence signalling pathways.

Bidirectional signal transduction between plants and inter–root microorganisms creates dynamic ecological association networks known as plant–microbe interactions. By controlling plant root development, nutrient uptake, antiretroviral gene expression, and other factors, microorganisms affect host adaptations, and plants generate selective pressures through the chemical gradient in their root secretions [40,41,42] (e.g., amino acids, organic acids, sugars, and alcohols, etc.). This interaction system accomplishes synergistic evolution through metabolic regulation. Through the chemical gradient of chemical pressure and the morphological plasticity of root hairs, the plant maintains system stability and controls the functional composition and geographical distribution of symbiotic flora [44]. Material cycling and energy flow in soil ecosystems are driven by this reciprocal relationship: plant litter and root secretions give microbial communities metabolic substrates, and greater microbial diversity prevents the growth of harmful bacteria by interspecies competition, thereby fostering benign soil microbial succession [45]. Vegetation–microbial synergism optimized the soil physicochemical properties, creating an ecological feedback mechanism of “root development-bacterial community activation–geotechnical improvement” (Figure 3 and Figure 4). Typical studies [46] have demonstrated that plant root expansion significantly increases soil enzyme activities by enhancing the release of inter–root secretions.

Alfalfa demonstrates multi-level symbiotic network formation traits, making it a model plant for studies of legume–microbe interactions. The phosphorus transport efficiency can be greatly increased by its mycelial network developed with an arbuscular mycorrhizal fungus (AMF), and the rhizobia-formed symbiotic system increases the host’s capacity to use nitrogen through nitrogen-fixing metabolism. Notably, the alfalfa root system secretes certain compounds (such as gibberellin, salicylamide, etc.) that may activate the systemic stress response pathway by causing the production of PGPR functional genes [48]. In addition to offering a theoretical framework for understanding plant–microbe co-evolution, a thorough examination of the interactions with mycorrhizal fungi, PGPR, and other microorganisms will set the stage for the development of biofortification technologies and the targeted control of inter–root microbial communities. These studies offer novel approaches for raising forage yield and quality and boosting agricultural system sustainability by elucidating the mechanism of microbial-mediated soil improvement. This is particularly useful for the bioremediation of salinized soil and the development of eco-agriculture.

### 3.2. Alfalfa–Microbe Interaction Mechanism and Its Effects

**Figure 5:**
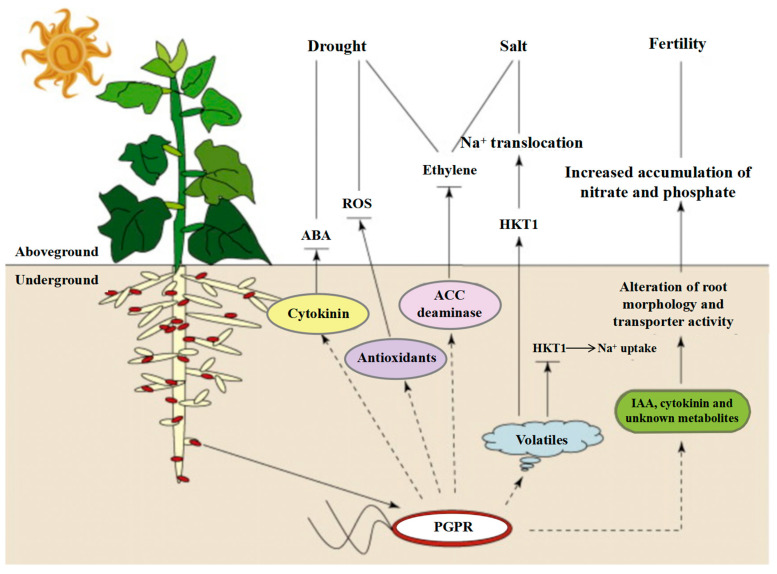
IST elicited by PGPR against drought, salt and fertility stresses underground (root) and aboveground [49]. Abbreviations: ABA, abscisic acid; ACC, 1–aminocyclopropane–1–carboxylate; HKT1, high–affinity K transporter 1; IAA, indole acetic acid; IST, induced systemic tolerance; PGPR, plant–growth–promoting rhizobacteria; ROS, reactive oxygen species.

The development of bidirectional metabolic control mechanisms between the host and the symbiont is at the heart of the synergistic evolutionary network that is made up of plant–microbe interactions. This network is built on material exchange and signaling. By secreting phytohormones and growth regulators, microorganisms in the alfalfa–microbe interaction system contributed to the development of the host root while also enhancing the plant’s resistance to adversity by activating the antioxidant system and osmoregulatory pathways. It is important to note that the flavonoids released by alfalfa roots create a particular chemical gradient. This inter-root metabolome feature not only offers functional microorganisms ecological niche selection pressure, but it also preserves the symbiotic system’s homeostasis by modifying the structure and metabolic activity of bacterial colonies in response to alkaline and saline stress. The combined action of AMF and PGPR has been shown to have a substantial ecological advantage. By altering the host ABA signaling pathway and activating the ROS scavenging system, PGPR decreased the physiological harm caused by adversity stress in alfalfa [49]. At the same time, its secreted IAA–like compounds improved the plant’s Na^+^ homeostatic balance by controlling the expression of the HKT ion-transporting proteins. Together, they provide a multilevel stress barrier, with this physiological regulating impact serving as a functional complement to the phosphorus transport mechanism mediated by the AMF mycelial network [50,51]. The study also showed that by improving the efficiency of inter–root carbon and nitrogen metabolism cycling, the interaction between PGPR and AMF may bi-directionally regulate soil enzyme activities and encourage microecological function remodeling [52,53,54,55] (Figure 5). In contrast, the symbiotic relationship between alfalfa and nitrogen–fixing bacteria represents a distinct process of nutritional reciprocity. Through the ammonium nitrogen assimilation pathway, nitrogen–fixing bacteria supply the host with nitrogen nutrition. The intermediates of the dicarboxylic acid cycle generated during their metabolism serve as energy substrates to replenish the nitrogen–fixing enzyme activity of the bacteria [56,57]. Through gene expression reprogramming, nitrogen–fixing bacteria under saline stress trigger the ammonium secretion response pathway. This adaptive evolutionary strategy not only preserves the symbiotic system’s nitrogen metabolism balance but also increases the nutritional value of alfalfa by controlling the phytosynthesis pathway [58,59,60]. By increasing the synthesis of osmoregulatory chemicals, salinity-tolerant rhizosphere-promoting strains have been shown in recent investigations to considerably increase the physiological adaptability of alfalfa roots under salt stress [61]. This mutualistic system’s ecological value is found in its multifaceted environmental effects: on the one hand, it increases plant resistance to accumulate biomass, and on the other hand, it regulates inter–root processes to promote soil organic matter synthesis and nutrient cycling. A theoretical framework for the creation of microbiome–regulated eco–agriculture models is provided by the impact of alfalfa–microbial synergism on improving soil structure, particularly the bioremediation capability of salinized soils. To effectively translate the theory of plant–microbe interactions into agricultural practice, future research must concentrate on the synergistic metabolic network analysis of functional strains and their methods for maintaining field stability.

## 4. Current Status of PGPR Research

Through a multifaceted regulatory mechanism, PGPR, a beneficial bacterial group that is advantageously colonized in the microecological system of the plant root system, supports crop growth and development. The synthesis of growth hormone, gibberellin (GA), and other phytohormones to promote the development of root morphology is the primary mechanism of action. The physiological processes of phosphorus solubilization, potassium solubilization, nitrogen fixation, and certain pathways to release soluble potassium and phosphorus for crop uptake and utilization to regulate the nutrient utilisation rate, in order to satisfy the plant’s nutrient demand, are the second primary mechanism of action [62]. Third, to reorganize the micro-ecosystems between the roots. Increase crop tolerance to abiotic stresses, such as salt stress, by improving the structure of the soil between roots by secreting compounds like extracellular polysaccharides [60,63,64,65,66,67,68,69,70].

In summary, PGPR employ multiple mechanisms to enhance plant growth and salt tolerance in saline–alkali environments. These include regulating phytohormone levels, reinforcing stress resistance pathways, improving soil conditions, and stimulating antioxidant enzyme activity. Additionally, PGPR secrete organic acids to lower soil pH, thereby facilitating plant adaptation and survival under stressful conditions [71,72]. Research demonstrates that PGPR synthesize various phytohormones to promote plant growth. For instance, they produce indole–3–acetic acid, a key auxin that stimulates root and shoot development [73], along with gibberellins and cytokinins, which collectively regulate plant growth processes [74]. Furthermore, PGPR enhances osmotic stress tolerance by synthesizing osmolytes like proline, thereby assisting plants in maintaining cellular water balance under stress conditions [75]. In addition to secreting extracellular polysaccharides (EPS) that promote soil aggregation and enhance water retention capacity [73,76], PGPR produce the enzyme ACC deaminase. This enzyme degrades the ethylene precursor ACC, thereby reducing stress-induced ethylene accumulation in plants and preventing its associated growth inhibition [73,76]. Furthermore, PGPR contribute to ionic homeostasis by regulating the Na^+^/K^+^ ratio in plant tissues, which reduces sodium toxicity and supports normal physiological functions [77]. They can also immobilize harmful heavy metals in the soil through biomineralization, thereby lowering metal bioavailability and alleviating phytotoxicity [78]. Additionally, the secretion of organic acids by PGPR helps regulate soil pH and improve soil physicochemical properties [72]. Another key mechanism involves the induction of antioxidant enzymes–such as superoxide dismutase (SOD), peroxidase (POD), and catalase (CAT)–which scavenge ROS generated under salt stress, mitigate oxidative damage, and collectively establish a more favorable rhizosphere environment for plant growth [79,80,81,82]. It is particularly noteworthy that to mitigate alkaline stress, PGPR primarily regulates soil pH and improves soil physicochemical properties by secreting organic acids [83].

Given the significance of PGPR in sustainable agricultural growth, the following five aspects will be the main topics of discussion in this paper: (1) genetic evolution and taxonomy based on genomics; (2) identification and isolation of functional metabolites and analysis of their mode of action; (3) the control network and mechanism of inter–root associative nitrogen fixation; (4) ecological investigation of colony origin and colonization dynamics; and (5) PGPR formulation research and development and application in adversity agriculture. The creation of green planting systems and the creation of novel microbial fertilizers will both benefit from the thorough investigation of these research avenues.

By means of multi-pathway synergistic effects, PGPR improve the environmental adaptability of their host plants. Their primary mechanisms include the regulation of ethylene metabolism, which is mediated by ACC deaminase, as well as synergistic networks of phytohormone synthesis, iron–carrier secretion, phosphorus activation, etc. While indole ethanol and indole acetic acid can both successfully decrease plant growth through the breakdown of ethylene precursors, ACC deaminase may effectively ameliorate the growth inhibition of plants caused by adversity. Growth inhibition through the breakdown of ethylene precursors, whereas the production of plant growth regulators, like IAA, directly encourages biomass accumulation and root conformation optimization [84]. By regulating the inter–root micro-ecology, certain strains of Bacillus subtilis, for example, can drastically alter the composition of the inter–root protist community and encourage the enrichment of functional microbial taxa, which in turn improves the effectiveness of phosphorus and the efficiency of nitrogen metabolism. This pleiotropic effect of PGPR in the alfalfa system is not only evident in the improvement of stress tolerance but also the remodeling of soil function [17,41,85].

PGPR has systemic ecological effects, which include optimizing plant physiology at the individual level through hormone signaling networks, rearranging inter–root functional modules at the community level through microbial interactions, and promoting soil nutrient cycling and energy flow at the ecosystem level. According to recent research, PGPR–mediated alfalfa–microbial interactions can synchronize the enhancement of biomass, the enhancement of protein synthesis, and the maintenance of soil health. The mechanism of action involves the regulation of bidirectional feedback between microbial metabolites and root secretion [86,87]. PGPR has shown great value in the bioremediation of degraded soils and the synergistic enhancement of crop resilience through the optimization of strain compositions and the improvement of delivery systems, despite the ecological competition of indigenous microbial communities in field application. This offers a theoretical foundation and a technical pathway for the development of a sustainable production system of leguminous pasture grasses.

### 4.1. Current Status of PGPR Genetic Classification Research

The functional characteristics of PGPR are significantly positively correlated with its genetic diversity, and its phylogenetic classification serves as both the fundamental starting point for identifying the molecular mechanism of plant–microbe interactions and the foundation for screening effective functional strains. The microbial phylogenetic tree is built using multilevel taxonomic ordinal elements, and the genetic categorization method of PGPR is founded on genomic traits and phylogenetic data. Research has indicated that contemporary microbial genetic classification technology has created a multi-omics matrix. For example [88,89,90,91], Yuanke Su et al. showed that 16S rRNA gene sequencing, the gold standard, has the primary benefits of high resolution, high species identification capability, standardized phylogeny, and high quality of microbes. Cuizhu Chen’s team also systematically summarized the application scenarios of 16S/18S rRNA gene sequencing, transcribed spacer region (ITS) analysis, whole–genome sequencing, and macro-genome sequencing, among others. With its primary benefits of high resolution, standardized sequence database support, and cross-species comparability, 16S rRNA gene sequencing is the gold standard and is especially well–suited for phylogenetic investigation of nitrogen–fixing bacteria. The major phylogenetic families of PGPR are now known to be Proteobacteria, Actinobacteria, Acidobacteria, and Bacteroidetes. This result is backed by genomic evidence from multiple studies, including Jones K.M. and others. Evidence in favor of this. It is important to note that research on alfalfa inter–root microbiomes still has a lot of unanswered questions, particularly in the area of genetic diversity resolution, which requires improvement. Rhizobial symbiotic systems, such as the unique relationship between alfalfa and *Sinorhizobium meliloti*, have garnered a lot of interest as a model system for legume–microbe interactions because of the following traits: (1) a high degree of conservativeness of symbiotic signaling pathways; (2) a high biological nitrogen fixation capacity, and (3) a host-specific identification mechanism. Symbiotic signaling pathway conservation. Alfalfa is a valuable research subject for examining the mechanism of inter–root microbial–plant co-evolution because of these traits. As a result, the genetic classification of alfalfa inter–root microorganisms can theoretically support the development of a microbiome–based sustainable alfalfa cultivation system in addition to clarifying the ecological origin and phylogenetic status of the core nitrogen–fixing bacteria.

### 4.2. Current Status of PGPR Metabolite Classification Research

By increasing the bioavailability of nutrients, PGPR directly encourage plant absorption and use. Phosphorus solubilization, potassium solubilization, and nitrogen fixation are among the physiological functions of PGPR. They release soluble phosphorus and potassium and dissolve soil insoluble phosphorus–potassium compounds by secreting organic acids and enzymes. At the same time, they reduce atmospheric nitrogen to ammonia through the nitrogen–fixing enzyme system to meet crop nitrogen requirements. This multi–pathway nutrient activation mechanism promotes crop growth and development and greatly increases the efficiency of plant nutrient uptake [62]. (1) Phosphorus solubilization. Microorganisms synergistically promote soil phosphorus solubilization through multiple pathways, firstly, microorganisms assimilate NH_4_^+^ releasing substances during ammonium assimilation, which leads to a decrease in soil pH and accelerates phosphate mineral dissociation; secondly, CO_2_ produced by microbial respiration dissolves in water to form carbonic acid and dissociates, which enhances phosphorus bioavailability through acidification of the environment; furthermore, hydrogen sulfide (H_2_S) produced by phosphorus–solubilizing bacteria metabolism interacts with phosphorus in the iron-binding form (Fe–P, such as FePO_4_) undergoes a reduction reaction (2FePO_4_ + 3H_2_S → 2FeS + S + 2H_3_PO_4_), which directly releases soluble phosphate ions (PO_4_^3−^) [3,92]. Together, these three mechanisms constitute a microbially driven phosphorus activation network. (2) Solubilization of potassium. By forming a complex with silicate minerals and adsorbing organic acids and inorganic ions with the aid of ionic, hydrogen, and covalent bonds, potassium–solubilizing bacteria work in concert to release mineral potassium through a variety of mechanisms. In the meantime, the polysaccharides specifically capture SiO_2_, disrupting the dynamic equilibrium of mineral dissolution–crystallization and speeding up potassium solubilization by altering the chemical environment of mineral microdomains. Second, the bacterially generated organic acids (such as citric and oxalic acid) separated and liberated H^+^, which reduced the pH locally, dissolved the aluminosilicate matrix on the mineral surface, and markedly increased the K^+^ solubility. The fixed state K^+^ is also released when mycelium growth occurs between mineral layers, causing mechanical forces that directly break the lattice structure of multilayer silicates like mica [93]. These three routes work together to create an effective activation network for bacteria that solubilize potassium.

**Figure 6:**
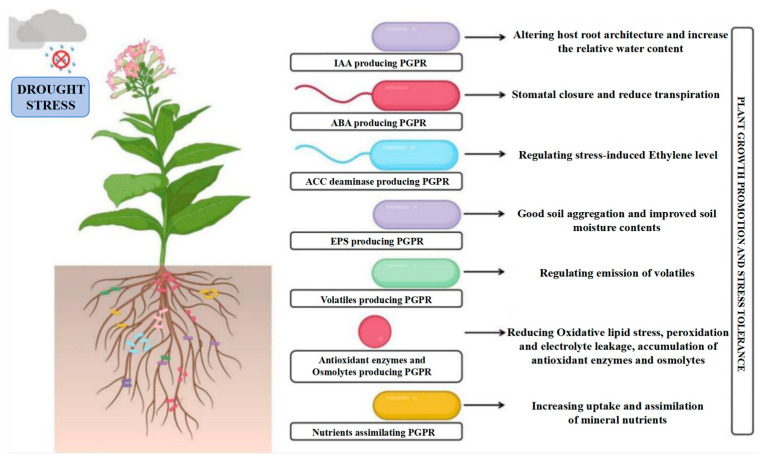
Graphical overview of PGPR functions in supporting plant growth and resistance to drought stress [98].

Through the Metabolomics Toolbox, PGPR metabolites—functional secondary metabolites generated during the strain–host plant symbiosis (Figure 6) [88]—play a triple synergistic role in promoting plant growth, inducing stress tolerance, and improving the inter–root microenvironment. PGPR strains can be categorized based on their secretion characteristics, according to functional classification studies based on metabolomics:Nutrient activation type: increasing the bioavailability of mineral elements by a variety of means, including the production of metal chelators, the secretion of organic acids, the activation of proton pumps, and enzyme–catalyzed reactions, such as the activation networks for iron, phosphorus, and nitrogen that Gou Yuchun’s group discovered ([88]); (2)The cytokinin (CTK), IAA, and gibberellin (GA3) synthesis pathways, for example, have been shown by Sabaté DC et al. to improve nutrient uptake efficiency by controlling the balance of endogenous hormones in plants [94]; (3) Soil amelioration—Etesami H et al. demonstrated that this strain can secrete extracellular polysaccharides, biofilm matrix, and other substances, which can significantly improve the soil granular structure and porosity and simultaneously boost the vitality of the root system [91]. For plant–microbe interactions, this metabolism–driven “inter–root dialogue” process serves as a two–way signaling system.

PGPR metabolic engineering exhibits multifaceted application value in the green alfalfa growing system: (1) Biological control dimension: Bacillus subtilis secretes the lipopeptide antibiotics iturin A and fengycin, which can disrupt the cell membrane sterol structure of pathogenic fungi (like *Fusarium oxysporum*) and kill them (inhibition rate of up to 70%). This can be used to stop the pathogenic fungus from growing. By disrupting the sterol structure of the cell membrane of harmful fungus, such as *Fusarium oxysporum*, an inter–root immunological barrier is established (70% inhibition rate) [95]; (2) Resilience enhancement dimension: under saline and alkaline stress, the proline content of PGPR–induced alfalfa was increased from 1.7902 to 295.84 times higher than that of the control, and nitrogen fixation enzyme activity was increased from 1.7902 to 295.84 times. (3) Nutritional enhancement dimension: inoculation treatments increased alfalfa’s phosphorus utilization from 15% to 35%, and the cumulative increase in stem and leaf biomass was up to 22% [96]. The rhizobial colonization density rose by 18.45%, while nitrogen–fixing enzyme activity increased to 4.79 times that of the control [97]. The metabolite pool of the alfalfa inter–root microbiome may contain novel plant immune activators (like lipopeptide inducers) and soil remediation agents (like heavy metal chelating proteins), in addition to the core functions of nitrogen fixation and stress tolerance. These should be investigated immediately using metabolome–transcriptome combination analysis. The development of “designed microbial fertilizers” and accurate control tools for the building of alfalfa low–carbon agriculture systems will be aided by this type of research.

**Figure 7:**
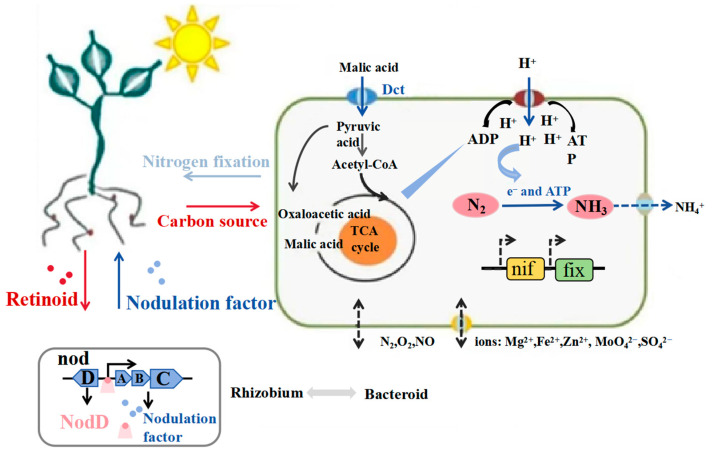
Mechanisms of rhizobia–plant host symbiosis [103] The symbiosis between rhizobia and the legume is initiated by the legume exudation of flavonoids, and they are recognized by rhizobial NodD. With binding to the flavonoids, NodD proteins are activated to induce the transcription of nodulation genes, such as nodA, nodB and nodC. Nod factors are synthetize to respond back to the host. Finally, the nodules form and rhizobia differentiate into bacteroids. In *bacteroids*, nitrogen fixation is processed under the cooperated regulation of rhizobial genes, metabolism and multiple internal environmental homeostasis.

More than 65% of the reactive nitrogen supply in the global ecosystem comes from biological nitrogen fixation, which is the main driver of the nitrogen cycle on Earth [99]. The reduction of atmospheric nitrogen to ammonia is catalyzed by nitrogen–fixing microorganisms through a nitrogen–fixing enzyme complex (NifHDK). The basic reaction can be expressed as follows: N_2_ + 8H^+^ + 8e^−^ + 16MgATP → 2NH_3_ + H_2_ + 16MgADP + 16Pi [85]. Nitrogen–fixing bacteria can be divided into three main functional groups according to their symbiotic traits (Table 1) [91,100,101]. Symbiotic nitrogen fixation microorganisms (e.g., *Rhizobium*–*legume symbiosis*), which form particular rhizo–cytoplasmic symbioses with leguminous plants; and autotrophic nitrogen–fixing bacteria (e.g., *Cyanobacteria*), which independently perform nitrogen fixation through photosynthetic phosphorylation or oxidative phosphorylation and are distributed throughout freshwater, marine, and terrestrial ecosystems. Associative nitrogen–fixing bacteria, like *Azospirillum*, accomplish nitrogen fixation by non–specifically colonizing plant root surfaces or cortical interstitial spaces, and their efficiency is in the middle of the first two. They also contribute more than 60% of terrestrial biological nitrogen fixation each year [102]. Due to the intricate molecular dialogue mechanisms involved (such as cascade control of the nodulation gene nodABC) (Figure 7) [103], the symbiotic nitrogen fixation system between rhizobia and legumes has emerged as a classic model for researching plant–microbe co–evolution.

When exposed to saline stress, salt–tolerant nitrogen–fixing bacteria help hosts restore ionic homeostasis by synthesizing compatible solutes (like ectoine) and antioxidant enzyme systems. Alfalfa is a pioneering saline land–amelioration crop. At the level of NCR peptides, nodule–specific cysteine–enriched peptides secreted by legumes (like MtNCR211) induce rhizobial differentiation through membrane potential interference, inducing symbiosis formation [104] alfalfa, a pioneer crop for improving salinity, has made significant strides in the study of its nitrogen fixation system. Liu’s team [105] isolated and screened 80 strains of endophytic bacteria, with an average bacterial inhibition rate of 66.11%, yielding the Bacillus–like bacteria MS–43 (*Paenibacillus* sp.). According to field tests, co–inoculation of *Pseudomonas fluorescens* and *Sinorhizobium meliloti* enhanced alfalfa crude protein content by 18% and nitrogen accumulation by 25% (up to 4.8 g/kg DW). Three main research bottlenecks still exist, though: (1) a lack of progress in building a germplasm repository of salt–tolerant nitrogen–fixingC; (2) a lack of thorough analysis of the network of multi–colony interactions; and (3) an unidentified mechanism governing the regulation of inter–root nitrogen–fixing functional gene expression. The development of a saline alfalfa–microbial symbiotic restoration system will receive crucial technical support if these scientific issues are resolved.

### 4.3. Current Status of Research on the Sources of PGPR

The chemotaxis gradient of the root secretion is closely linked to the ecological preference of PGPR in plant inter–root ecosystems, which exhibit spatial heterogeneity in their distribution and population densities along the “intra–root → root surface → inter–root soil → non–inter–root soil” [106], where the number of microorganisms in the root system is 100–1000 times higher than that in the non–root system. A gradient that is strongly related to [106]. Research has revealed notable variations in the distribution patterns of various functional PGPR: (1) The nitrogen–fixing flora *Rhizobium* (*Rhizobium*, *Bradyrhizobium*, etc.) symbiotically binds to legumes and fixes nitrogen biologically by forming specialized organs called rhizomes on the roots [107]; (2) Tang et al. [108] demonstrated that dephosphorylating strains (like *Pseudomonas*) activate insoluble phosphates by secreting phosphatases, phytases, and low molecular weight organic acids. For instance, *Pseudomonas* produces a lot of organic acids that fix aluminum by chelation, making it less harmful to plant roots in extremely worn soils and increasing the solubility of phosphorus [109]. (3) Despite being of low order of magnitude, intra–root colonizing bacteria (such as Bacillus subtilis) are important physiological regulators through systemic induced resistance (ISR) [110]. In essence, this three–dimensional distribution network, known as “inter–root–root surface–intra–root,” is a spatial metabolic division of labor where microbes and plants co–evolve.

#### 4.3.1. Research on Inter–Root Endophytes

**Figure 8:**
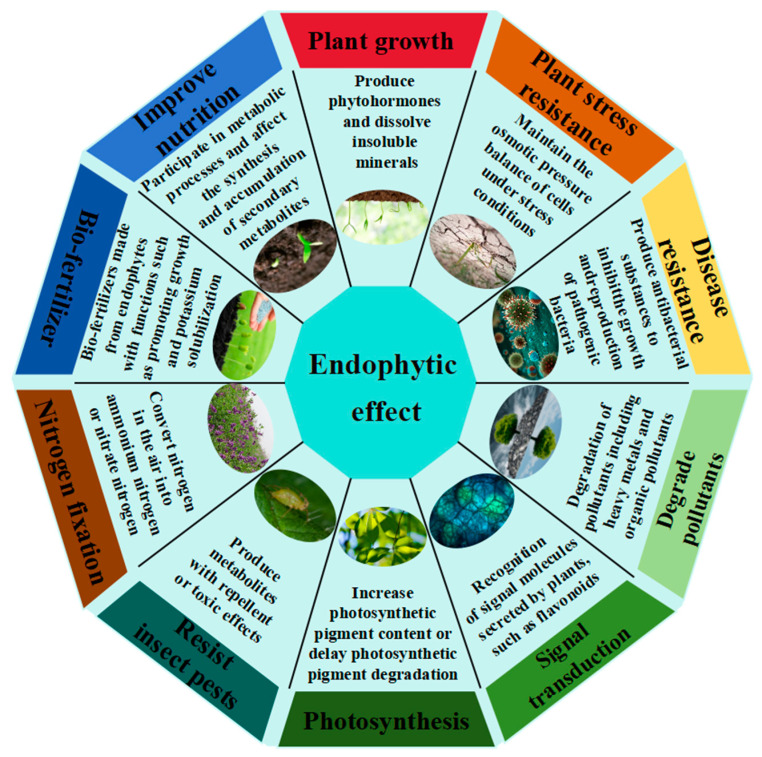
The role of endophytes in plants.

**Figure 9:**
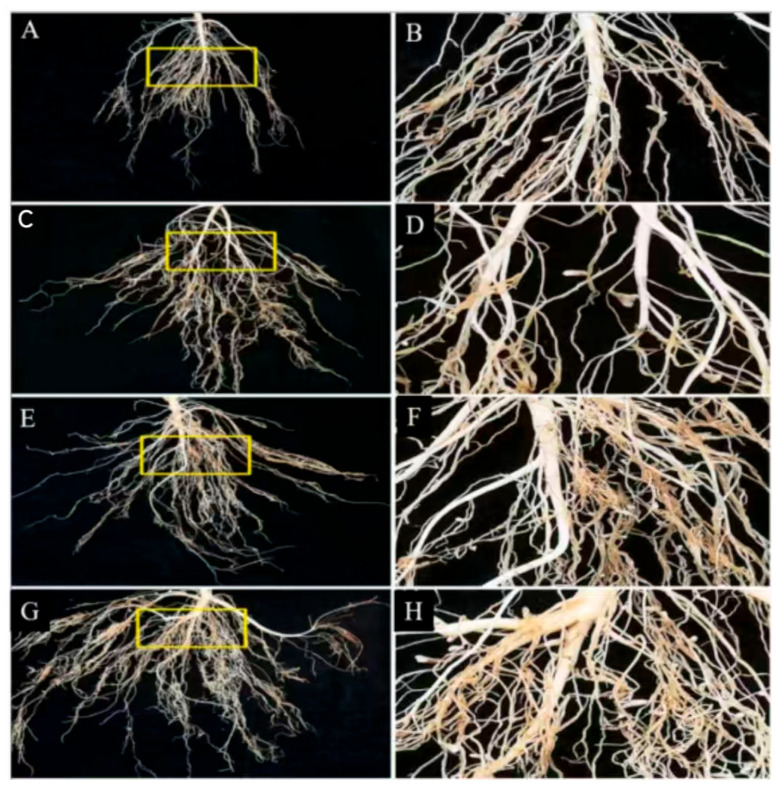
Roots of un–inoculated bacteria (**A**,**B**) and inoculated with MJM–11 (**C**,**D**), GL1 (**E**,**F**), and “GL1 + MJM-11” (**G**,**H**) treatments. Yellow rectangles show the nodules in the roots of the treatments more clearly [97] (*Enterobacter ludwigii* MJM–11 and *Sinorhizobium meliloti* GL1).

By establishing metabolic connections that colonize many tissues, endophytic bacteria, which are “natural biofactories” in plant microcosms, contribute in multiple ways to plant growth, development, and adversity adaptation (Figure 8). The “two–bacteria symbiosis system” created by Hong Gao’s group is particularly noteworthy [97]; under 200 mM NaCl stress, the co–inoculation of salt–tolerant *Enterobacteriaceae* MJM–11 and Chinese alfalfa *Rhizobium meliloti* GL1 produced a threefold synergistic effect: (1) Ionic homeostasis regulation: the plant can play a multifaceted regulatory role in growth, development, and adversity adaptation by establishing a metabolic mutualistic network and cross–tissue colonization (Figure 9). (1) control of ion homeostasis: the root tip’s Na^+^ content was decreased by upregulating the expression of the SOS1 and NHX1 genes; (2) Nitrogen fixation enhancement: the activity of the nitrogen fixing enzyme was enhanced to 2.01 nmol C_2_H_4_/g/h, which is 378.57% of the initial value; and (3) symbiotic enhancement: the density of *rhizobium* colonization rose by 18.45%. Field tests verified that this approach considerably decreased soil salinity, raised dry matter yield by 26.12%, and boosted alfalfa crude protein content from 18.13% to 22.54%. According to these studies, PGPR not only uses the “metabolite–gene–microbiome” three–level regulatory network to accomplish the traditional biotrophic function, but it also exhibits special benefits in saline–alkali restoration: its synthesised lipopeptides, such as surfactin, improve the health and productivity of the plant by activating the MAPK signaling pathway, and its secreted ACC deaminase can break down stress ethylene precursors. To improve plant systemic resistance, use the MAPK signaling pathway. To create carefully controlled “plant–microbe co–evolution” improvement strategies, future studies must concentrate on the mechanism of endophyte transport across tissues and its interactions with host epigenetic alterations.

#### 4.3.2. Research on Inter–Root Soil Bacteria

**Figure 10:**
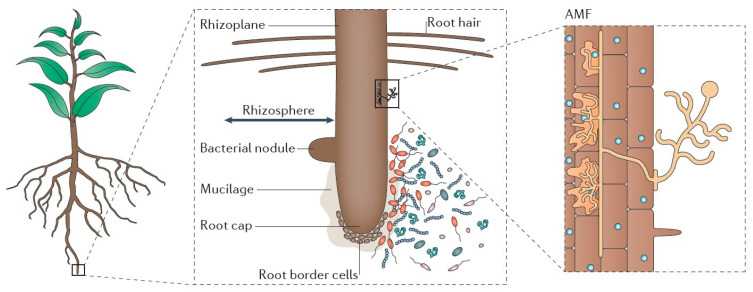
Inter–root soil, microbial, and secretion interactions [113] The schematic shows magnified pictures of the rhizosphere, containing saprophytic and symbiotic bacteria and fungi, including AMF.

**Figure 11:**
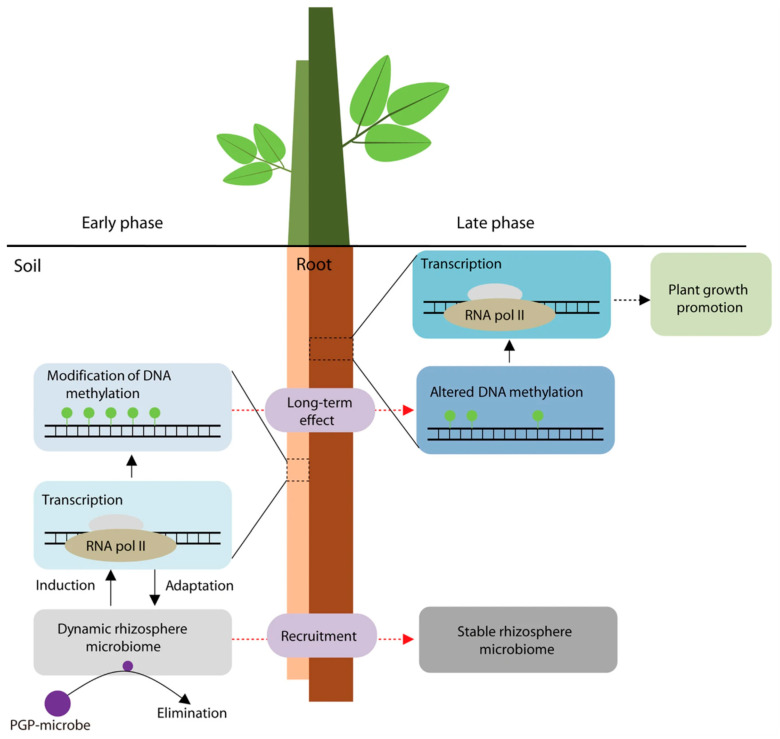
Diagrammatic illustration of the two–step process by which DNA methylation and root recruitment mediate the interaction between PGPB and plants. The PGPB inoculum is represented by purple circles. Methylated cytosine is represented by green circles [112]. In the early phase, inoculation with PGPB induced variation in the rhizosphere microbiome. Plants adapt to the dynamic rhizosphere microbiome through comprehensive changes in transcription profiles, including DNA methylation–related genes, which results in the modification of DNA methylation. The influences of inocula on the rhizosphere microbiome weaken along with the elimination of the inoculum from the rhizosphere microbiome. In the late phase, the altered DNA methylation regulates gene expression to facilitate plant growth, and a stable rhizosphere microbiome is assembled by recruitments of roots.

Microorganisms that live in or colonize plant roots are known as inter–root soil bacteria. They can help plants acquire mineral nutrients and enhance their growth. By controlling the dynamics of inter–root microbial biomass, Li et al. [111] discovered that the chemical constituents of root secretions affect the variety of microbial communities (Figure 10). For instance, under salt stress conditions, PGPR dramatically improved the nitrogen fixation efficiency of the *rhizobium*–*alfalfa* symbiotic system by favorably regulating the rhizoma growth and nitrogen fixing characteristics of the system. From the inter–root of alfalfa in the petroleum hydrocarbon–contaminated area, Sergey N. Golubev’s team obtained *Mycolicibacterium* sp. PAM1, a strain of the *Mycobacterium* family, and shown to significantly boost the growth of the hosts. The significance of DNA methylation changes in roots in response to plant growth–promoting bacteria (PGPB) was a more intriguing discovery. Chen Chen et al. suggested a novel mechanism for PGPR–induced root DNA methylation modifications to stimulate plant growth. Their findings demonstrated that the promotion process was mediated by PGPB–induced root DNA methylation modifications, which continued to be effective even after the inoculum was removed from the microbiome [112] (Figure 11).

**Figure 12:**
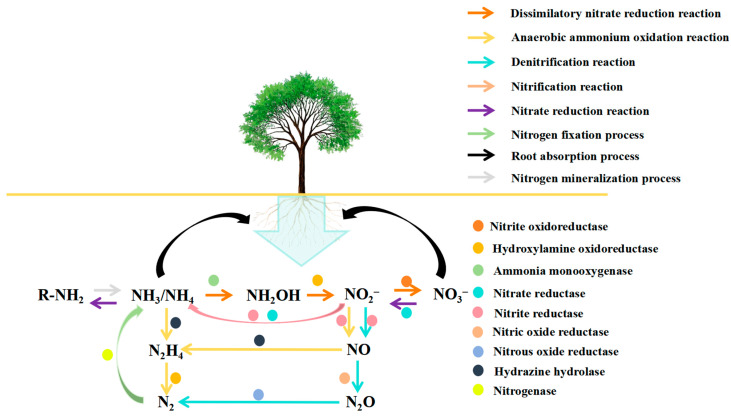
Important inter–root microbial activities related to the soil nitrogen cycle [117].

According to current research, the main functional flora of plant inter–roots are nitrogen–fixing bacteria such as *Azotobacter*, *Rhizobium*, and *Arthrobacter* [114]. Among these, nitrogen–fixing bacteria use biological nitrogen fixation to transform atmospheric nitrogen into ammonium nitrogen, which significantly improves the plant’s resistance to low–nitrogen stress [115]. Cui Yue’s study group [116] found that a range of highly nitrogen–fixing active strains existed in the inter–root of alfalfa, and the host root secretion may drive the enrichment of particular strains. The main paths of inter–root microorganisms involved in the soil nitrogen cycle are methodically shown in Figure 12. Together, the aforementioned study findings support the crucial function that alfalfa inter–root nitrogen–fixing microbes play in nitrogen transformation.

### 4.4. Applied Research on PGPR

**Figure 13:**
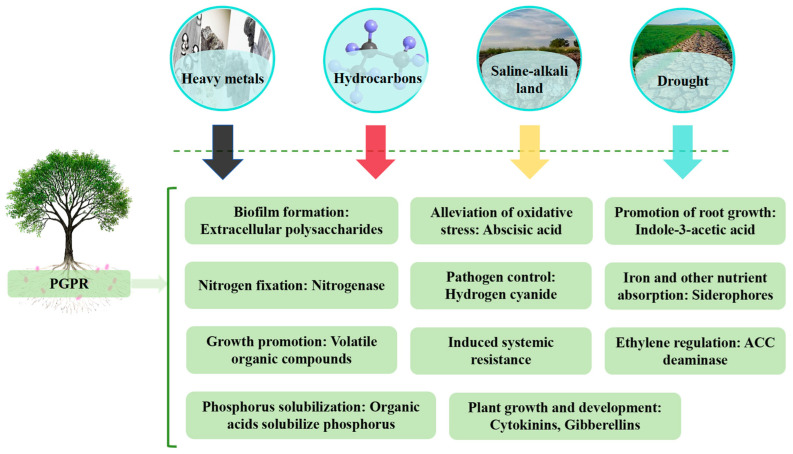
Application of PGPR to soil [119] Abbreviations: EPS (exopolysaccharides); ABA (abscisic acid); IAA (indole acetic acid); HCN (hydrogen cyanide); VOCs (volatile organic compounds); ACC (1–aminocyclopropane–1–carboxylic acid).

High–throughput sequencing and soil physicochemical property analysis are among the research approaches used to study plant–derived PGPR, which have significant application value in soil remediation and geotechnical enhancement [114,118]. By boosting soil nutrient content and enzyme activity, PGPR controls plant nutrient intake and developmental processes, as seen in Figure 13. It also serves an ecological remediation role in the management of contaminated water bodies.

#### 4.4.1. Application of PGPR in Soil Improvement

In the realm of remediating damaged soil, alfalfa exhibits special benefits. In contrast to conventional remediation methods, Ma et al. [120] demonstrated that PGPR–mediated soil amelioration can more effectively preserve the natural qualities of soil. It should be noted that because of their high toxicity and challenging decomposition, high molecular weight polycyclic aromatic hydrocarbons (HMW PAHs, ≥4 tightly packed aromatic rings) have emerged as a significant environmental issue brought on by oil spills and incomplete fuel combustion [121]. These pollutants have created ecological hazards in a number of places [122,123,124,125], and because of its affordability and environmental friendliness [126,127], combined plant–microbe remediation technology has emerged as a key approach to modern pollution control. Alfalfa is a fast–growing perennial plant with a deep root system that can bind and break down PAHs through trichomes and offer ecological niches for microorganisms. The plant may be a natural home for bacteria that break down HMW PAHs, such as Mycobacterium spp., according to studies [128,129,130]. Using the plant’s inter–root–adapted degrading bacteria can greatly increase the effectiveness of phytoremediation. By controlling soil microbial activity, PGPR can greatly increase the effectiveness of phytoremediation. By controlling the soil microbial community, preventing acidification, and increasing the rate of nutrient conversion, PGPR enhances the plant microenvironment. Its nitrogen–fixing strains have also been used as biofertilizer [131]. Soil fertility can be directly improved by ammonium nitrogen, which is created during the nitrogen fixation process [132].

#### 4.4.2. Application of PGPR in the Plant Growth Promotion Mechanism

**Figure 14:**
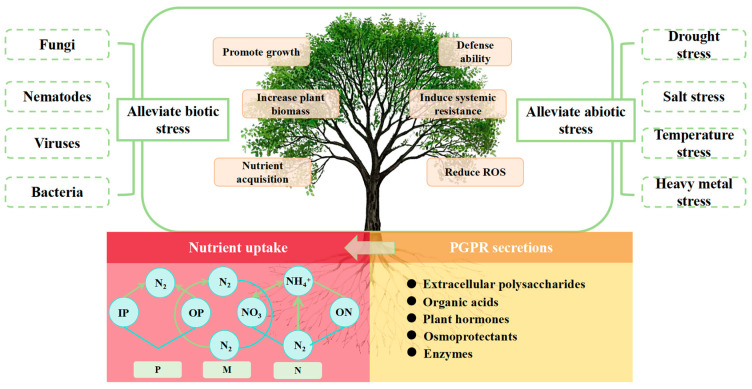
Schematic diagram of the mechanism of PGPR to promote plant growth [88] (EPS: extracellular polymer; AP: available phosphorus; IP: insoluble phosphorus; OP: organic phosphorus; EPS: extracellular polymer; SM: soluble minerals; IM: insoluble minerals; ON: organic nitrogen; M: mineral elements, e.g., potassium, iron, zinc, etc.).

Much emphasis has been placed on the regulating mechanism of PGPR on plant tolerance in environments of salt stress. By encouraging the manufacture of metabolites, strains like Bacillus subtilis can improve plant salt tolerance [133], which aids in the adaptation of plants to saline conditions [134]. According to Nabil Tirry et al. [17], PGPR can enhance mycorrhizal colonization and soil enzyme activity while reducing the harm that salt stress causes to alfalfa roots. Studies by Chen et al. [61] and Sharma et al. [135] further confirmed that PGPR promotes growth in saline soils. Furthermore, through metabolic processes like potassium and phosphorus solubilization, nitrogen fixation, and phytohormone synthesis [136,137] (Figure 14), PGPR activate soil nutrients. Its secretion of iron carriers and antifungal metabolites (e.g., hydrolases and HCN) can also effectively fend off pathogenic microorganisms [138,139].

#### 4.4.3. Application of PGPR in Ecological Environment Remediation

**Figure 15:**
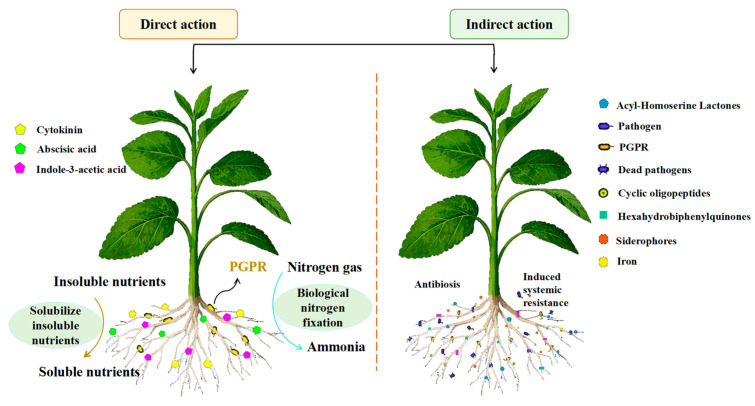
Direct and indirect mechanisms promoting host PGPR interactions and plant development [119].

By means of metal passivation mechanisms, oxidative defense, and osmotic regulation, PGPR improves plant chemical resistance in the management of heavy metal pollution [131,140]. Alfalfa’s root secretions, which enhance nutrient cycling, encourage microbial growth, and facilitate metal chelation, are intimately linked to its resistance to heavy metals [126,132,133]. By controlling ethylene synthesis, activating the antioxidant system, and secreting iron carriers, PGPR dramatically improved plant tolerance to heavy metal stress, according to the study [127,141,142] (Figure 15). Alfalfa infected with PGPR continued to grow well in the heavy metal environment, as demonstrated in the pot experiment [143]. PGPR exhibits two possible applications in the fields of heavy metal pollution remediation and salty land improvement: microbial–assisted phytoremediation offers a fresh perspective on heavy metal pollution management, and salt–tolerant strains can increase crop yield in saline conditions. For instance, PGPR and biochar together can maximize the nitrogen use efficiency and soil microbial community structure, as well as theoretically support precision fertilization [144]. Exogenous phosphorus addition trials and the screening of copper–tolerant PGPR strains showed that they improved alfalfa growth performance in copper–contaminated soil and reduced the toxicity of Cu^2+^ stress by increasing soil enzyme activities (sucrase, etc.) [145].

Recent research has revealed significant advances in understanding how PGPR enhances the ecological restoration and sustainable use of soda–alkaline soils. Empirical studies demonstrate that co–inoculation with PGPR and rhizobia alters the diversity and composition of the rhizosphere protist community. Concurrently, this approach reduces soil saline–alkali properties and increases nutrient content, collectively establishing a more favorable environment for plant growth under stress [146]. Beyond their use in formulating biofertilizer consortia for sustainable crop production under drought stress [147].

PGPR also enhance crop tolerance to soda–type saline–alkali stress by modulating the rhizosphere microbiome. For instance, inoculation with *Bacillus halotolerans* has been shown to enrich beneficial microbes such as *Pseudomonas*, *Sphingomonas*, *Klebsiella*, and *Bacillus*, while suppressing plant pathogens. This shift in microbial community structure helps plants maintain growth and viability under adverse conditions [12,13,14].

## 5. Conclusions and Outlook

Together, the inter–root and endophytic nitrogen–fixing microorganisms of alfalfa plants–a highly effective symbiotic nitrogen–fixing bacterial community—play a crucial role in nitrogen delivery, despite the plants’ limited ability to fix nitrogen on their own. The nitrogen–fixing strains identified from this taxon are notable for their ability to both promote plants and withstand stress, underscoring the scientific significance of studies on alfalfa’s inter–root–endophytic nitrogen–fixing bacterial resources. Alfalfa is employed extensively in salty soil improvement as a pioneer plant with excellent nitrogen fixation efficiency and salinity tolerance; yet, little is known about its endophytic and inter–root nitrogen–fixing microbiota. To provide theoretical support for the research and development of new biological fertilizers and ecological restoration of saline and alkaline soils, we must systematically analyze the diversity of inter–root nitrogen–fixing bacteria in saline and alkaline habitats in the future. We also need to clarify the molecular mechanism of biological nitrogen fixation and the regulatory network of environmental adaptation. Furthermore, by integrating plant–microbe interactions, the synergistic adaptation mechanism between nitrogen–fixing bacteria and alfalfa may be further examined, potentially leading to new avenues for the sustainable use and biological enhancement of saline soils.

## Figures and Tables

**Table 1:** Three major types of nitrogen–fixing microorganisms.

Biological Nitrogen Fixation System		Types of Nitrogen–Fixing Microorganisms
Free living nitrogen fixation microorganisms	Phototrophs	*Anabaena*, Green sulfur bacteria
	Chemolithotrophs	*Leptospirillum ferrooxidans*
	Heterotrophs	Aerobic: *Azotobacter* *Facultatively* anaerobic: *Klebsiella*. Some *Bacillus* spp. Anaerobic: *Clostridium*, *Methanogens*
Symbiotic Nitrogen Fixation Microorganisms		*Rhizobium-legume* symbiosis *Rhizobium-Parasponia* symbiosis *Frankia-dicotyledon* (non–legume) symbiosis Diazotrophic cyanobacteria-plant symbiosis
Associative Nitrogen Fixation Microorganisms		*Azospirillum*, *Azotobacter*, Some *Pseudomonas* spp.

## Data Availability

No new data were created or analyzed in this study. Data sharing is not applicable to this article.
